# 391. Evaluation of oral vancomycin treatment for hospital-acquired *Clostridioides difficile* infection prophylaxis in a community hospital: A retrospective cohort study

**DOI:** 10.1093/ofid/ofac492.469

**Published:** 2022-12-15

**Authors:** Qingqing Meng, Heather Cohen, Bidhya Poudel, Pabitra Adhikari, Nagwa Abou-Ghanem, Paritosh Prasai, Valiko Begiashvili, Kritika Yadav, Emre C Ozcekirdek, Guillermo Rodriguez Nava, Guillermo Rodriguez Nava

**Affiliations:** Ascension Saint Francis Hospital, Evanston, Illinois; Ascension Saint Francis Hospital, Evanston, Illinois; Ascension Saint Francis Hospital, Evanston, Illinois; Ascension Saint Francis Hospital, Evanston, Illinois; Ascension Saint Francis Hospital, Evanston, Illinois; Ascension Saint Francis Hospital, Evanston, Illinois; Ascension Saint Francis Hospital, Evanston, Illinois; Ascension Saint Francis Hospital, Evanston, Illinois; Ascension Saint Francis Hospital, Evanston, Illinois; Stanford University School of Medicine, Palo Alto, California; Stanford University School of Medicine, Palo Alto, California

## Abstract

**Background:**

Hospital-acquired (HA) *Clostridioides difficile* infection (CDI) is among the most common hospital-acquired infections and is a leading cause of morbidity and mortality among hospitalized older adults. Oral vancomycin prophylaxis (OVP) has been demonstrated in recent studies to reduce the incidence of HA CDI. This study aims to evaluate the effectiveness of OVP in the prevention of HA CDI in a community hospital setting.

**Methods:**

We developed a protocol to administer prophylactic oral vancomycin based on patients’ risk factors and implemented it at our community hospital in Evanston, Illinois, in September 2020. The intervention group consists of patients admitted to our hospital between October 1, 2020, to March 31, 2021, who received OVP. Patients admitted between October 1, 2019, to March 31, 2020, who had at least one risk factor of CDI and met the inclusion criteria of OVP protocol were selected as the control group. Electronic medical records were retrospectively collected and analyzed. Propensity score matching was performed for a one-to-one match between two groups. Logistic regression models were used to study the relationship between HA CDI and independent variables, including OVP, risk factors of CDI, and demographic characteristics.

**Table 1.**
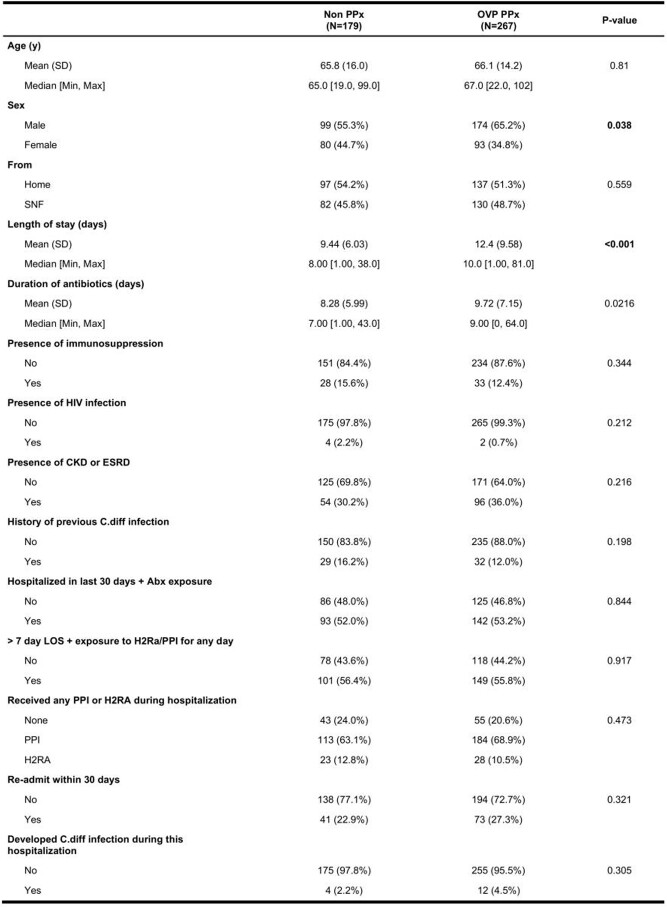
Sample characteristics of the control and intervention groups

A Student t-test was performed for continuous parameters. A Chi-square test was performed for categorical parameters. Non PPx = control group; OVP PPx = oral vancomycin prophylaxis group; SNF = skilled nursing facility; LOS = length of stay; PPI = proton pump inhibitor; H2RA = histamine 2 receptor antagonist.

**Results:**

A total of 446 patients (267 in the intervention group and 179 in the control group) were included. The sample characteristics are summarized in **Table 1**. 4/175 (2.2%) patients in the control group developed HA CDI, compared with 12/255 (4.5%) in the intervention group. Before matching, patients who received OVP (OR=7.62, *p*=0.010) and had a longer length of stay (LOS, OR=1.11, *p*=0.002) were found to have increased odds ratios (OR) of development of HA CDI (**Table 2**). After propensity score matching, 4/176 (2.3%) patients in the control group developed HA CDI, compared with 5/176 (2.8%) (**Figure 1**). Patients from skilled nursing facilities (SNF, OR=15.41, *p*=0.021) and with longer LOS (OR 1.15, *p*=0.005) were found to be associated with higher OR of HA CDI (**Table 3**).

**Table 2.**
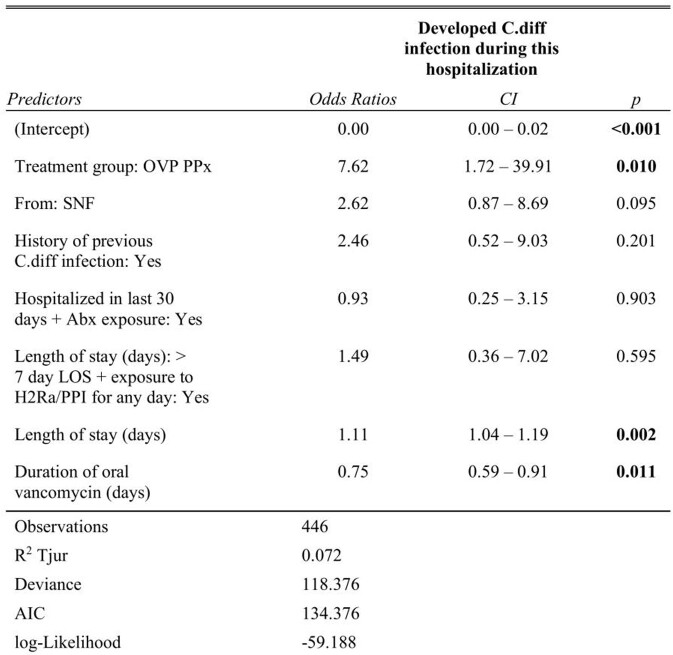
The odds ratio of development of hospital-acquired C.diff infection among control and intervention groups.

A binary logistic regression model was used for this study. CI = 95% confidence interval. R2 Tujur = coefficients of determination (D); AIC = Akaike’s Information Criteria. OVP PPx = oral vancomycin prophylaxis group; SNF = skilled nursing facility; LOS = length of stay; PPI = proton pump inhibitor; H2RA = histamine 2 receptor antagonist.

**Figure 1.**
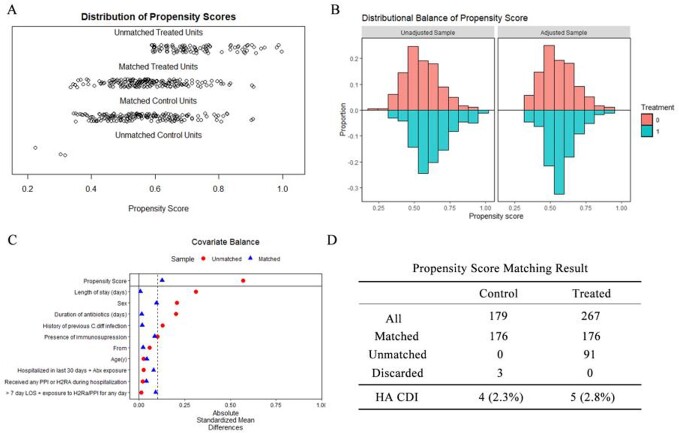
Propensity score matching of control and intervention groups.

(A) Distribution of propensity scores between unmatched and matched control and intervention groups. (B) Distribution balance of propensity score before and after matching. Treatment o = control group; Treatment 1 = OVP intervention group. (C) The distribution balance of covariates used for propensity score calculation before and after matching. (D) Summary of propensity score matching results. HA CDI = hospital-acquired C.diff infection (after propensity score matching).

**Table 3.**
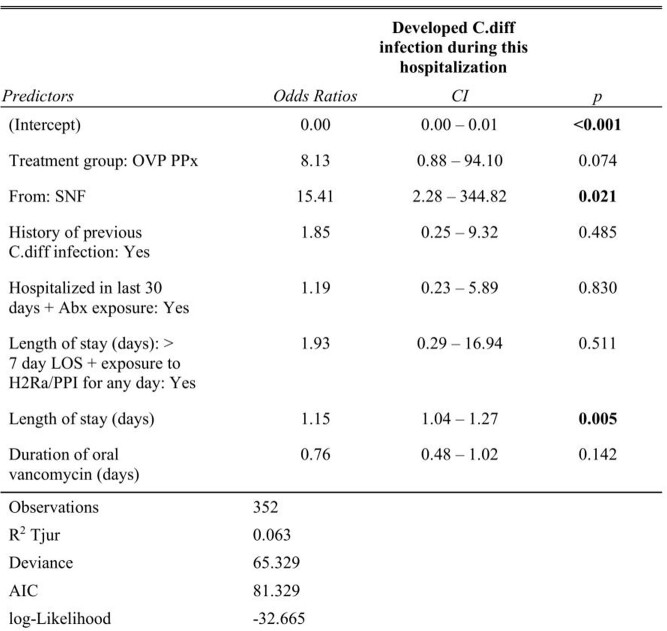
The odds ratio of development of hospital-acquired C.diff infection among control and intervention groups after propensity score matching.

A binary logistic regression model was used for this study. CI = 95% confidence interval. R2 Tujur = coefficients of determination (D); AIC = Akaike’s Information Criteria. OVP PPx = oral vancomycin prophylaxis group; SNF = skilled nursing facility; LOS = length of stay; PPI = proton pump inhibitor; H2RA = histamine 2 receptor antagonist.

**Conclusion:**

Prophylactic administration of oral vancomycin to patients with selected risk factors has no statistical significance in reducing or preventing HA CDI. A longer length of hospital stay may be associated with higher risk for developing HA CDI.

**Disclosures:**

**All Authors**: No reported disclosures.

